# Single cell–inductively coupled plasma–mass spectrometry (SC-ICP-MS) reveals metallic heterogeneity in a macrophage model of infectious diseases

**DOI:** 10.1007/s00216-024-05592-3

**Published:** 2024-10-17

**Authors:** Claire Davison, Jordan Pascoe, Melanie Bailey, Dany J. V. Beste, Mónica Felipe-Sotelo

**Affiliations:** 1 School of Chemistry and Chemical Engineering, Faculty of Engineering and Physical Sciences, University of Surrey, Guildford, UK; 2 Department of Microbial Science, Faculty of Health and Medical Sciences, University of Surrey, Guildford, UK

**Keywords:** Single cell analysis, Cell fixation, Inductively coupled plasma mass spectrometry, *Mycobacterium tuberculosis*

## Abstract

Single cell–inductively coupled plasma–mass spectrometry (SC-ICP-MS) offers an attractive option for rapidly measuring trace metal heterogeneity at the single cell level. Chemical fixation has been previously applied to mammalian cells prior to sample introduction so that they can be resuspended in a solution suitable for SC-ICP-MS. However, the effect of fixation on the elemental composition of suspended cells is unknown, and robust methodologies are urgently needed so that the community can measure the effects of intracellular pathogens on elemental composition of their host cells. We demonstrate that different fixatives impact measured cell elemental composition. We have compared suspensions treated using different fixatives (methanol 60–100% in H_2_O and 4% paraformaldehyde in phosphate-buffered saline solution), and the number of distinguishable single cell events, keeping a constant particle number concentration. Significantly more single cell events (*n* = 3, *P* ≤ 0.05) were observed for Ca and Mg when cells were fixed in 4% paraformaldehyde than for the methanol-based fixatives, confirming the hypothesis that methanol fixatives cause leaching of these elements from the cells. The impact of fixation on Mn and Zn was less pronounced. Microbial and viral infection of eukaryotic cells can have profound effects on their elemental composition, but chemical fixation is necessary to render infected cells safe before analysis. We have successfully applied our methodology to a macrophage model of tuberculosis demonstrating utility in understanding metal homeostasis during microbial infection of mammalian cells.

**Graphical Abstract:**

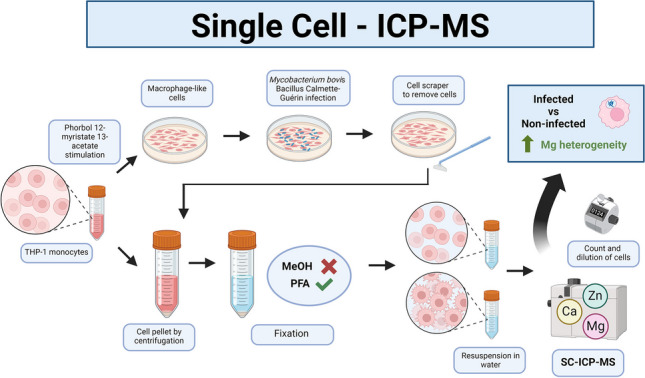

**Supplementary Information:**

The online version contains supplementary material available at 10.1007/s00216-024-05592-3.

## Introduction

Metal homeostasis has a critical role in the immune response and has therefore become an important focus in the field of immunometabolism [1, 2]. Bulk analysis techniques are frequently used to measure trace metals and provide insight into responses occurring at the cellular level [3, 4]. The disadvantage in studying average analyte concentrations from cells cultures is that heterogeneity, which is shown to have an impact on many biological processes, is masked [5]. Time-resolved single particle–inductively coupled plasma–mass spectrometry (SP-ICP-MS) is able to analyse single cells in suspension (SC-ICP-MS) and therefore measure this heterogeneity that is invisible to bulk measurements. This has been successfully applied to a number of biological, environmental and medicinal chemistry studies [6, 7].

Sample preparation is critical to SC-ICP-MS measurements but remains poorly investigated. The media required for mammalian cell culture is highly complex (including proteins, lipids, amino acids, and inorganic salts [8]), and hinders the identification of single particle/cell ionisation events from the matrix background. Direct introduction into the instrument is therefore not possible. Chemical fixation is required to resuspend mammalian cells in an alternative medium that is more compatible with ICP-MS (e.g. water) by preserving intracellular components and cellular structures, which results in a better signal to noise ratio [9, 10]. This chemical sterilisation is also an essential step for studying eukaryotic cells infected with microbes, so that analytical measurements can be carried out safely [11, 12].

Methanol (MeOH) [13, 14] and paraformaldehyde (PFA) [15] are commonly used to fix mammalian cells prior to SC-ICP-MS analysis; however, the effect of these fixation procedures on the measurement of cell elemental composition is unknown. Prior research has focused on the effect of fixatives on the elemental composition of tissues rather than single cells. For example, Hare et al*.* [16] showed that aldehyde fixation results in the leaching of metals (K, Mg, Fe, Cu and Zn) from murine tissue into the fixative solution, as observed as well by Gellein et al*.* [17] for formalin-fixed brain tissue. Leaching is thought to be the result of the different types of binding interactions between metals and biomolecules [18]. Efforts are therefore needed to characterise the impact of fixation on metals in cells. Furthermore, cellular applications of SC-ICP-MS have predominantly been focused on toxicological studies of metal/metalloid [19, 20] and nanoparticle [21, 22] uptake into algal [19] and yeast cells [21, 23]. These cells can be easily suspended into solutions with low background such as water. However, analysis of mammalian cells to study bacterial infections by SC-ICP-MS presents more challenges as these cells require very complex media to grow, are frequently attached to a surface and are vulnerable to osmotic lysis. Consequently, there are few studies which employ SC-ICP-MS to characterise mammalian cells [6, 14, 24, 25] and bulk analysis approaches are most often used to study immunological responses [3, 4, 26, 27]. One of the goals of this study was to develop a method of cell fixation prior SC-ICP-MS that was suitable for the handling and fixation of infectious microorganisms, which in turn would facilitate studies into elemental homeostasis and heterogeneity during the study of host cell/pathogen interactions.

Macrophages are white blood cells with functions that include the recognition and phagocytosis of foreign pathogens and production of pro-inflammatory chemokines and cytokines to control infection. THP-1 monocytes are a cancer cell line that can easily be differentiated into macrophage-like cells and are commonly used as a macrophage model system for various biological applications [28]. Here, we have developed a protocol to enable the comparison of the impact of aldehyde and alcohol fixatives on the concentrations of Mg, Ca, Zn and Mn in THP-1 cells. Mg and Ca were selected for these studies due to their high abundance in eukaryotic cells and established role in mitochondrial metal ion transport [29, 30]. Mn and Zn are transition metals that are not only essential for the survival of intracellular pathogens within their host cells but are also linked to the immune responses of host cells against various microbes [31]. The developed methodology was then applied to a macrophage model of tuberculosis, demonstrating for the first time the utility of SC-ICP-MS in a comparative study to understand metal homeostasis during microbial infection of mammalian cells. Importantly, although we developed this method with THP-1 macrophages, it could be applied to other mammalian cells and infection models.

## Materials and methods

### Cell culture

**Fig. 1:**
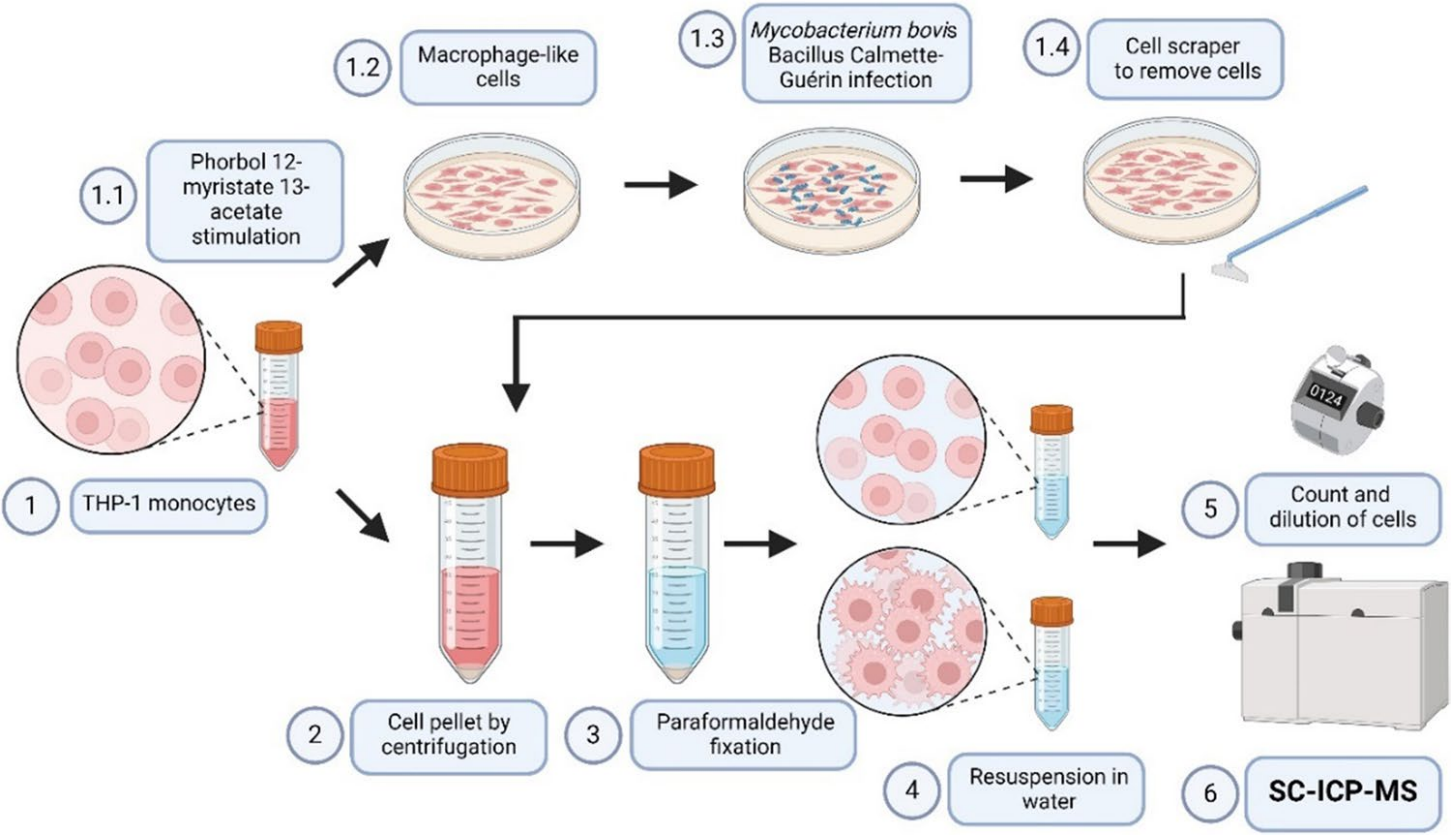
Sample preparation procedure used for the measurement of trace metals in single THP-1 monocytes, non-infected macrophages and BCG-infected macrophages using SP-ICP-MS

THP-1 (ATCC) cells were grown in vented tissue culture flasks and stored in an incubator at 37 °C with 5% CO_2_. Rosewell Park Memorial Institute (RPMI) 1640 cell culture media (Sigma) and 10% heat-inactivated fetal calf serum (FCS, from Sigma) were used to provide growth factors for the cells. Prior to SC-ICP-MS analysis, the cells were chemically fixed and resuspended in deionised water. Unless otherwise stated, 4% PFA in phosphate-buffered saline (PBS, Sigma) was used to fix the cells. For this, the cellular suspension was pelletised by centrifugation (Thermo Heraeus, Megafuge16R, 300 × g, 5 min) and the cell culture media removed. 1 mL of the PFA solution was added to the cell pellet and the pipette was actioned repeatedly to allow for gentle mixing and resuspension of the cell pellet, before being left for 30 min at room temperature. Following fixation, the cells were washed twice with PBS and once with deionised water to completely remove the fixative before being counted and diluted to the appropriate concentration (see Fig. 1, steps 1–6).

### Cell counting

Simple light microscopy using a standard Neubauer haemocytometer and the Trypan blue viability stain were used to count cells (Fig. 1, step 5). Trypan blue (0.4–0.8% solution, Sigma) was used to identify the number of live cells within a suspension in a process known as dye exclusion. Undamaged live cells do not uptake Trypan blue into the cytoplasm and therefore remain unstained. Dead and damaged cells uptake the negatively charged trypan blue molecule [32]. An aliquot of the cellular suspension of unknown concentration was dyed (1:1 ratio) and mixed gently before being pipetted into the counting chamber. Using this method, the total number of viable cells can be estimated with good accuracy and precision (< 10%) and three replicate counts were used for each experiment.

### Macrophage model of tuberculosis

THP-1 monocytes were activated using phorbol 12-myristate 13-acetate (PMA, Sigma) and infected with a multiplicity of infection (MOI) of 10 with *Mycobacterium bovis* Bacillus Calmette–Guérin (BCG) Pasteur as a surrogate containment level 2 model for the pathogen *Mycobacterium tuberculosis* [33]. For this work, we used a strain of *M. bovis* BCG: mCherry that contained an extra chromosomal plasmid constitutively expressing mCherry so that the bacteria could be visualised using fluorescent microscopy and flow cytometry. 5 × 10^5^ cells mL^−1^ were activated with 50 ng mL^−1^ of PMA for 36 h. THP-1 macrophages were then washed twice with PBS (with MgCl_2_ and CaCl_2_) before infection at a multiplicity of infection of 10 bacteria to 1 macrophage. Following incubation at 37 °C and 5% CO_2_ for 4 h, the cells were gently washed twice with PBS to remove extracellular bacteria before incubating for another 24 h in RPMI-1460 + 10% FCS. The cells were then carefully removed using a cell scraper (see Fig. 1, steps 1.1–1.4), collected, and fixed following the same procedure described above (Fig. 1, steps 1–6).

Flow cytometry analysis of BCG infected THP-1 macrophages was carried out with the Attune NxT flow cytometer (ThermoFisher Scientific, USA). The cells were processed in 1.5-mL Eppendorf tubes. To identify the ‘infected macrophages’ population, gates based on side-scatter area (SSC-A) for size confirmation and YL1-A (Ex 561 nm/Em 610 ± 15 nm) were established to detect fluorescence from the mCherry protein. Additionally, uninfected THP-1 cells were processed to exclude the ‘uninfected macrophages’ population based on the signal in the YL1-A channel. To further ensure accuracy, BCG mCherry bacteria was processed as a control to exclude fluorescence resulting from ‘free’ BCG present in the samples. This exclusion was based on size determination from SSC-A. During the analysis, a total volume of 450 μL at a flow rate of 200 μL min^−1^ was used, with a collection cutoff set at 5000 total events. Flow cytometry showed that a MOI of 10 resulted in approximately 25% of the macrophages being infected with bacteria.

### Instrumental conditions and optimisation of SC-ICP-MS analysis

Single cell analysis using time-resolved mode was carried out using the 7800 Series ICP-MS and Mass Hunter software (Agilent Technologies, UK). Unless otherwise stated, the default operating parameters are shown in Table SI 1. The background counts were distinguished from those produced by single cells using a Poisson statistics algorithm. The peaks over the threshold (≥ mean + 3 × SD) were considered to be single particle events and removed for further iterations, whereas all points below this limit were identified as background signal. This process was repeated until no data points above the threshold remained [34].

Transport efficiency (TE) is defined as the ratio of analyte entering the detector to the amount of analyte aspirated and is required to quantify trace metals in single particles [34]. There are two methods to determine TE, commonly referred to as the ‘pulse frequency method’ and the ‘particle size method’, described in full detail by Pace et al*.* [34]. Unless otherwise stated, the default approach to calculating TE was the ‘pulse frequency method’ using a cell suspension of a known particle concentration, counted using hemocytometry. Full details of assumptions and calculations of the transport efficiency using the ‘pulse frequency method’ can be found in the Supplementary Information. For the optimisation experiments, Mg was used as an endogenous elemental marker of the presence of single cells. This metal was selected due to its abundance in mammalian cells [29], which is reflected by the high signal to noise ratio of single particle ^24^ Mg events against the background of the sample matrix and the high number of single cell intensity peaks. This isotope was selected due to its high natural abundance and offered improved sensitivity compared to alternative magnesium isotopes, which is critical for single cell analysis [23, 35–37].

Finally, the SC-ICP-MS analysis procedure was compared to the average analyte concentrations in the cell suspensions determined by bulk analysis. A THP-1 suspension was fixed and counted to obtain a total of 10^7^ cells. The cells were then pelletised by centrifugation (Thermo Heraeus, Megafuge16R, 300 × g, 5 min), washed with PBS, and resuspended in 200 µL of deionised water. This was then added to 1 mL of the SC-hydrogen peroxide (30% in water, Fisher Chemicals) and 9 mL of nitric acid (trace metal grade 67–69% in water, Fisher Chemicals) and digested using the ETHOS UP™ microwave system (35 min, 1800 W, 210 °C). The resulting digests (*n* = 3) were filtered (syringe-top filters 0.22 µm, Millex-GP) and diluted using deionised water prior to analysis by ICP-MS. Mg (Fisher Scientific), Ca, Zn and Mn (PlasmaCAL) calibration standards were prepared in 2% nitric acid. Indium (100 ng/L) was used as the internal standard for all samples and calibration standards and good linearly was obtained for all four analytes in the range between 1 and 2000 ng L^−1^ (*r*^2^ > 0.986). The limits of detection (LOD) were 0.09, 2, 0.5 and 0.3 ng L^−1^ for Mg, Ca, Zn and Mn, respectively. A certified reference material (NIST 1643f) was used to determine instrumental accuracy, and for all 4 analytes, analytical recoveries ranged between 87 and 111% (*n* = 3).

## Results and discussion

### Instrumental optimisation

To optimise both preparation of the suspensions in terms of cell number concentration and operational conditions of the SC-ICP-MS (namely integration time, sample flow rate and nebuliser gas flow rate), a series of experiments were carried out to maximise TE and the number of peaks. Since the ﻿‘pulse frequency method﻿’ accounts for whole particle introduction rather than mass, using actual samples rather than reference materials (which are often not of comparable size and/or composition to the particles being analysed) is the most accurate representation of the behaviour of the real particles. Therefore, the ‘pulse frequency method’ using THP-1 cells was applied for the determination of the TE through the optimisation of the experimental conditions.

**Fig. 2:**
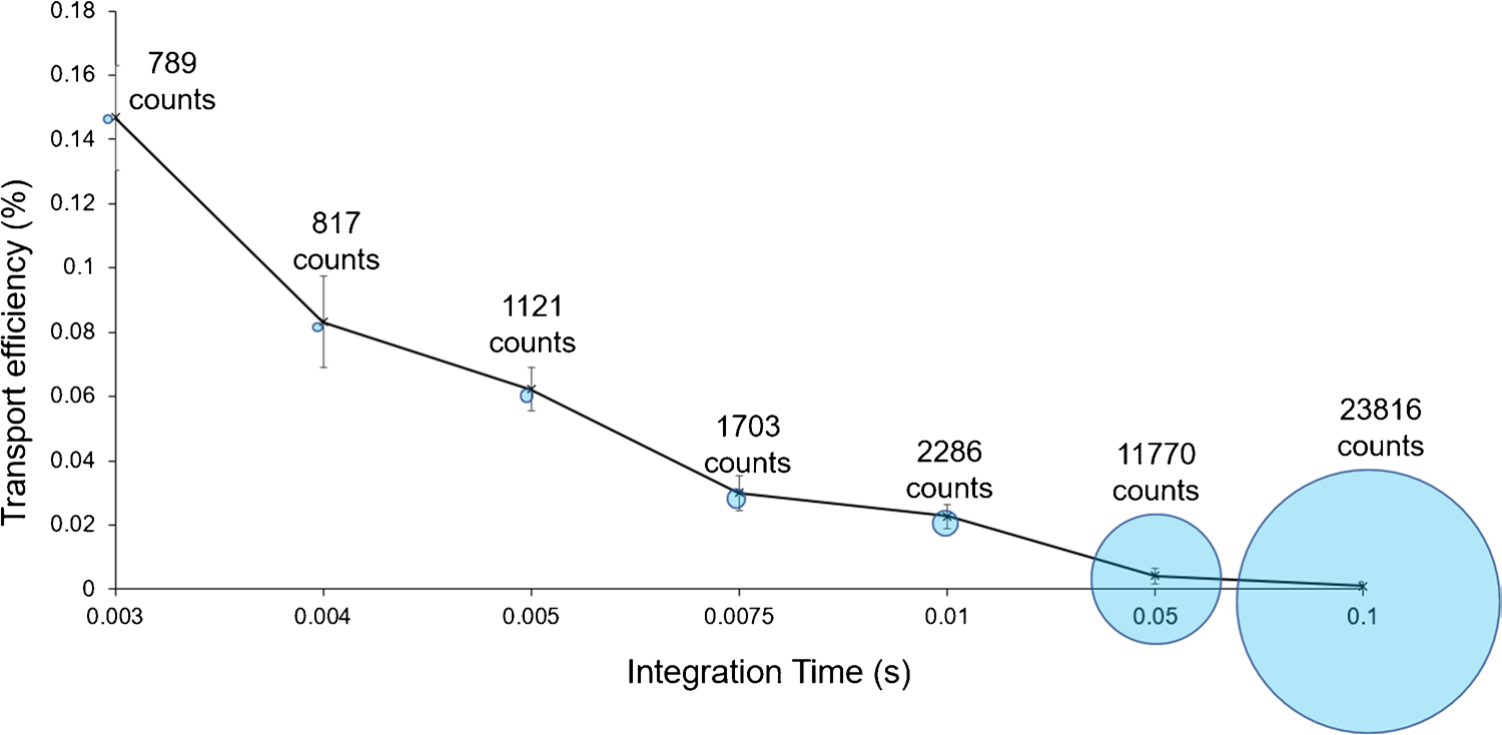
Transport efficiency (TE%) of THP-1 cells (calculated using.^24^ Mg peaks) analysed by SC-ICP-MS across varying integration times, with circles representing the maximum count intensity signal. Note the line between points does not represent a relationship between integration time and TE% and is included to guide the eye and highlight the decreasing trend. TE% has been calculated by the ﻿‘pulse frequency method﻿’ recommended by Pace et al. [34]. Error bars represent ± SD (*n* = 6)

A suspension of approximately 5 × 10^5^ cells mL^−1^ was prepared and analysed at integration times of 0.003, 0.004. 0.005, 0.0075, 0.01, 0.05 and 0.1 s, ranging from the shortest instrumental integration time available to a value at which peaks were no longer distinguishable from the background. The selection criterion for integration time was the highest number of detected intensity peaks attributable to single cell events. Figure 2 shows that TE decreased as wider integration windows were selected, while the maximum count intensity was found to increase with the integration time, illustrated by the circles overlapping the individual data points. At longer integration times the signals observed were most likely no longer pertaining to single cells and were instead being caused by the detection of multiple overlapping cells, reflected by the increase in maximum intensity counts. This is also supported by the linear correlation between the maximum count intensity and the integration time (*n* = 6, *R*^2^ = 0.995, see Fig. SI 1). Long integration times (50–100 ms) should therefore not be used for SC-ICP-MS, as illustrated by Fig. 2. Integration time of 0.003 s was selected as it fulfilled both criteria of producing intensity peaks attributable to single cell events and the highest TE.

**Fig. 3:**
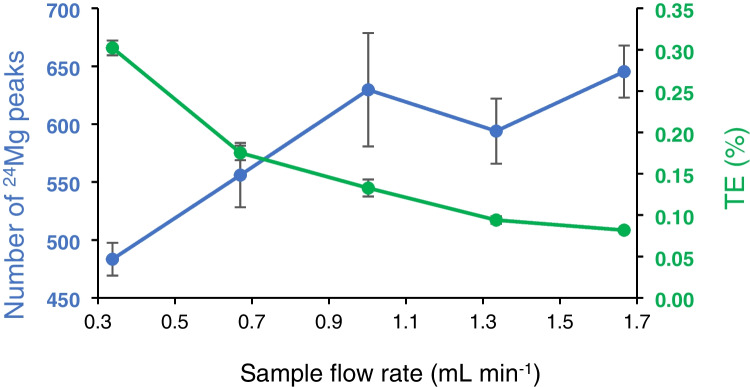
Sample flow rate (mL min^−1^) plotted against the number of distinguishable ^24^ Mg peaks and transport efficiency (TE%, ﻿‘pulse frequency method’﻿ [34]) obtained by SC-ICP-MS analysis of THP-1 cells. Note the line between points does not represent a relationship between sample flow rate, number of.^24^ Mg peaks and/or TE% and is included to guide the eye and highlight the trends. Error bars represent ± SD (*n* = 3)

The same THP-1 suspension was then analysed at sample flow rates from 0.3 to 1.7 mL min^−1^. The general trend showed an increase in single cell intensity peaks between 0.3 and 1.0 mL min^−1^ which plateaued between 1.00 and 1.7 mL min^−1^ when uncertainty of the measurements was considered. Whilst the number of peaks obtained increased with sample flow rate, TE dropped from 0.3 to 0.1% (Fig. 3), showing that the sample flow rate conditions required to obtain a high TE were not always those which provided the highest number of single cell ionisation events (1.7 mL min^−1^). TE calculated using the ‘particle frequency method’ is inversely proportional to the concentration of cells being introduced [34]. Therefore, a compromise should be reached, and the application of the method should determine whether a high TE or a high cell count should be prioritised. If limited sample volume is available (e.g. clinical samples) and/or absolute quantification may be required, then optimisation should be directed towards obtaining a high TE to make the best use of the available material. If the analyst has ‘unlimited’ access to the sample material (e.g. an immortal cell line) or in comparative studies, then obtaining a dataset with high statistical significance should be prioritised by increasing the sample flow rate or concentration of particles being introduced. However, an important consideration is the proportional increase in the total volume required for both stabilisation of the instrument and subsequent measurements resulting in increments of cost associated with reagents and analysis time. Stability of the plasma was also assessed by ensuring that the cutoff values for a cell and the peak maxima remained consistent across the different sample flow/peristaltic pump rates (Fig. SI 2). For a 1.5-mm injector torch, an optimal nebuliser gas flow rate was found to be 0.6 to 0.7 L min^−1^ with an increase of RSD found at flow rates exceeding this range (> 10%).

To determine the optimal particle number concentration, a series of cellular suspensions with concentrations ranging from approximately 5 × 10^3^ to 5 × 10^6^ cells mL^−1^ were analysed. Each suspension was counted before and after SC-ICP-MS analysis to ensure that the cell count remained stable between initial fixation and the analytical measurements. Cell counts were considered stable when found to be within ± 1 × SD (*n* = 3) of the original count after 12 h at room temperature (Table SI 2). The number of intensity peaks was found to increase from 10^4^ cells mL^−1^ to 5 × 10^5^ cells mL^−1^ and decrease between 10^6^ and 5 × 10^6^ cells mL^−1^. Excessively high cell number concentrations (≥ 10^6^ cells mL^−1^) led to an overlap of signals and an overestimation of the trace metal content of the cells.

### Optimisation of sample preparation

Once instrumental conditions and cell number concentration were optimised, focus was directed towards sample preparation. Direct analysis of a live THP-1 suspension in culture media was not possible because the complexity of the culture media was found to hinder the distinction of single cell peaks over the background signal. Blockages of the nebuliser also occurred with a higher frequency (Fig. SI 3–5). This demonstrates that cell fixation was an essential step in the analysis of mammalian cells.

MeOH [13, 14] and PFA [15] are often used in the literature for the fixation of cell suspensions, and therefore, we selected MeOH and PFA for further study. 70% MeOH in water and 4% PFA in PBS were initially tested. This resulted in some cell loss (assessed using a haemocytometer) with a greater drop in cell numbers observed in MeOH (− 27%, *n* = 3) than in PFA (− 12%, *n* = 3) when compared to the pre-fixed suspension. Moreover, the suspension fixed with PFA also had a higher TE with 0.10 ± 0.01% and 0.06 ± 0.01% for PFA and MeOH respectively. One possible reason for lower TE when using MeOH is the stability of the cell structure post fixation; if cell number concentration decreases between the initial count and analysis, then TE would be underestimated. However, the number concentration of cells, determined by hemocytometry, was found to be stable for both fixatives during a period of 12 h (Table SI 3). An alternative explanation is the occurrence of thinning, damage and/or leaching of the cell membrane caused by the MeOH. This would cause overall concentrations of metals (in this case the Mg used as an indicator of single cell events) to be lower, and therefore, the number of cells with sufficient analyte to be statistically above the background signal of the matrix also decreases. It has been previously reported that MeOH preserves intracellular structures poorly, and also cell membranes [38]. In contrast, aldehyde-based fixatives, such as PFA, provide improved preservation of the cellular structure compared to alcohol-based fixation agents and it is therefore the standard fixation method for a number of downstream analyses [39].

**Fig. 4:**
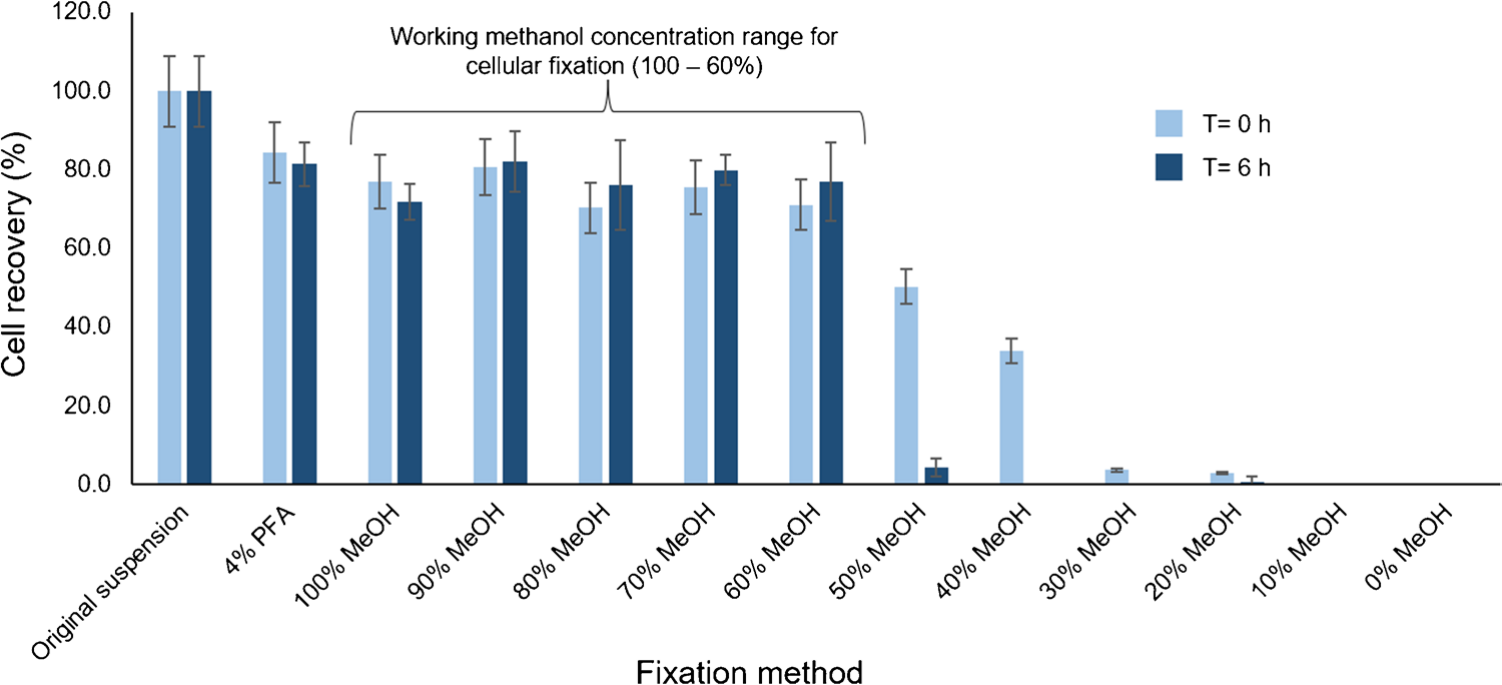
Cell recoveries counted using hemocytometry of 2-mL aliquots of a THP-1 suspension fixed using 4% PFA and 0 to 100% MeOH at *t* = 0 h and *t* = 6 h. Error bars represent ± SD (*n* = 3)

To assess this hypothesis of metal leaching induced during cell fixation, further SC-ICP-MS measurements were completed to evaluate the number and intensity of distinguishable peaks attributable to single cell events, under different fixation conditions and times but constant particle number concentration. We made the assumption that any decrease in the number of peaks would be a result of fewer cells containing sufficient analyte to be above the intensity cutoff for a single cell and therefore an indication of leaching. The working concentration range of MeOH fixation was determined using the criteria of ≥ 70% recovery of cells (assessed by hemocytometry) and a stable cell count within a 6-h time period. Using this criterion, the concentration range of MeOH was 60 to 100% (Fig. 4). Therefore, three concentrations of MeOH representing this range (60%, 80% and 100%) were selected and compared to 4% PFA in the SC-ICP-MS analysis.

**Fig. 5:**
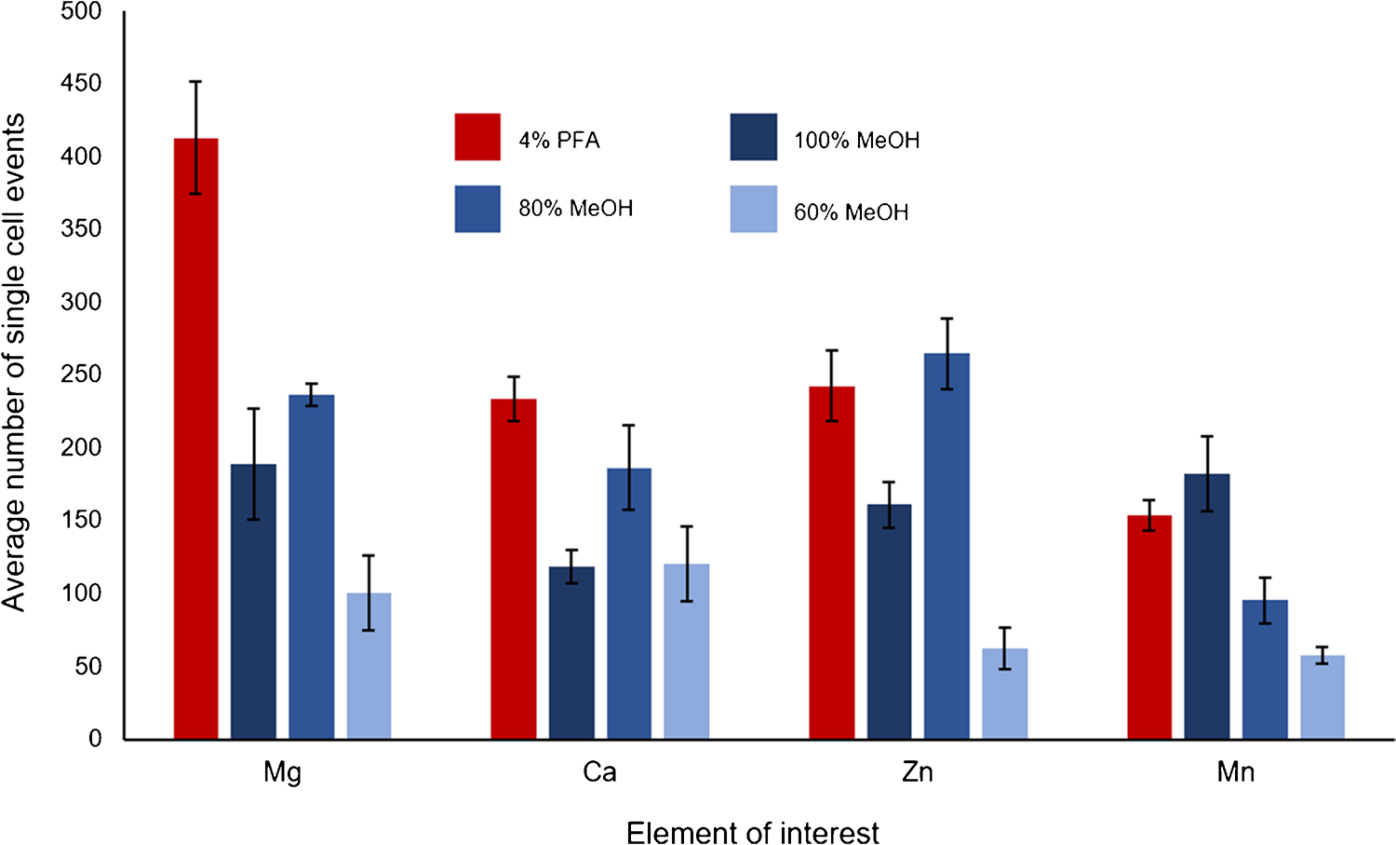
Number of intensity peaks pertaining to single cell events measured for ^24^ Mg, ^44^Ca, ^66^Zn and ^55^Mn using 4% PFA (red), 100% MeOH, 80% MeOH and 60% MeOH (blue). Error bars represent ± SD (*n* = 3) of the number of single cell intensity peaks detected during three 60-s time-resolved measurements

These experiments demonstrated that metal ion leaching was element dependent (see Fig. 5). Magnesium was the most severely affected by MeOH fixation, with percentage reductions in the number of single cell intensity peaks (55 ± 6%, 42 ± 7% and 75 ± 9% for 100%, 80% and 60% MeOH) in comparison to PFA (*n* = 3). A similar pattern was noted for calcium with reductions of 20–49% in MeOH fixed cells. This was not caused by cell viability issues as the number of viable cells was stable during this time period. This confirms that MeOH fixation impacts the analyte and also damages the cell membrane enough to facilitate Mg and Ca leaching from the cell. For zinc, 100% MeOH and 60% MeOH were inadequate for fixation as percentage reductions of the number of intensity peaks were high (33 ± 2% and 74 ± 4% respectively, *n* = 3) when compared with both 4% PFA and 80% MeOH. Mn leaching was minimal with 4% PFA and 100% MeOH whereas fixation with 80% and 60% MeOH resulted in significant leakage as indicated by reductions of 38 ± 8% and 62 ± 5%, respectively. These results demonstrate how important it is to select the appropriate fixation depending on the analyte being measured. In conclusion, 4% PFA was the most robust method of fixation that resulted in the least leaching. However, as PFA is quite a toxic compound, MeOH is a possible alternative depending on the analyte being measured. This was further reinforced by bulk measurement ICP-MS analyses. Levels of Mg and Ca were found to be significantly higher than those for Mn and Zn, for which the leaching effect was less severe (see Table SI 4).

### Comparison with bulk analysis

SC-ICP-MS analysis was compared to the average analyte concentration in the cell population with the bulk measurement after complete dissolution of the suspension (see “Materials and methods” section). The average mass per cell obtained using bulk measurement was found to be higher than the mass content from single cell measurements (full details on the calculation of the analyte mass content per cell and results can be found in Supplementary Information and Table SI 5), with relative errors ranging from 86 to 99% when using PFA fixation and TE ﻿‘particle frequency method’﻿ with THP-1 cells. While PFA was determined to be the optimal fixation method for the group of analytes of interest, PFA fixation could still be causing leaching and/or cell damage, only to a lesser extent than the MeOH leaching which was causing the discrepancy in the number of peaks detected. To investigate this further, the background Mg signal of a THP-1 suspension fixed using PFA at the optimal concentration of 5 × 10^5^ cells mL^−1^ was compared to the untreated water used to resuspend cells. The time-resolved background of the cell suspension was significantly higher (2474 ± 58 counts, *n* = 3) than the water blank used to resuspend the cells (14 ± 4 counts, *n* = 3). To pinpoint the cause of this increase in background signal, an aliquot of PFA fixed cell suspension in water was centrifuged (300 × g, 3 min) and the supernatant was collected, repeating the process until no intact cells or visible cellular debris were observed under the microscope. The resulting centrifuged supernatant (998 ± 214 counts, *n* = 3) presented lower counts than the THP-1 suspension background, and this decreased even further after filtration (0.22 µm) (22 ± 10 counts, *n* = 3). These results suggest that a significant proportion of the suspension background was being produced by cellular debris (≥ 0.22 µm in size) rather than continuously leached metals caused by PFA fixation.

As the concentrations of analyte being measured were in the low ng mL^−1^ region, even minor levels of cellular debris can result in a severe overestimation of the measured concentration per cell using a bulk measurement. Quantitative bulk measurements of fixed mammalian cells and the application of these techniques as a means of SC-ICP-MS validation should therefore be used with caution. Works by Mavrakis et al*.* [19] and Qin et al*.* [36] used algae and yeast cells respectively, without any fixation. Mavrakis et al*.* [19] reported masses of 2.67–4.07 fg for SC-ICP-MS compared to 3.2–7.2 fg for bulk measurements of As uptake and Qin et al*.* [36] reported good accuracy with only 20% underestimation by SC-ICP-MS for endogenous Mg, P, K, Mn and Cu. In SC-ICP-MS, the presence of dissolved debris is accounted for in the time-resolved spectra by correcting against the sample-specific background signal. Single cell measurements therefore offer a more accurate insight into trace metal levels compared to bulk techniques. However, one of the limitations of SC-ICP-MS is that the analyte concentration within a portion of the cell population may be too low to be detected using this methodology.

### Macrophage model of tuberculosis

**Fig. 6:**
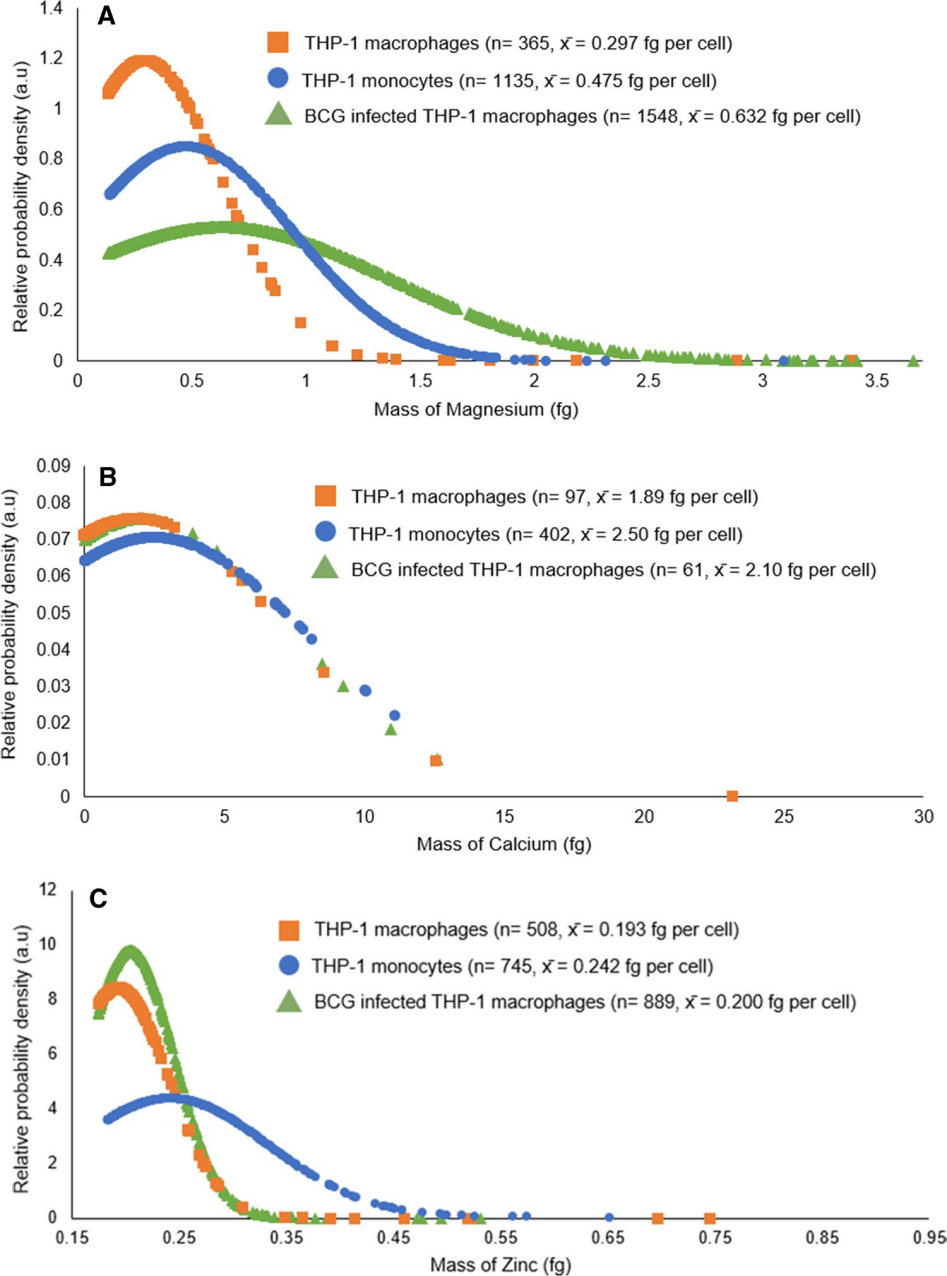
Normalised probability distributions of Mg (**A**), Ca (**B**) and Zn (**C**) in THP-1 monocytes, uninfected macrophages and BCG-infected macrophages. Probability densities shown are based on the accumulation of all single cell intensity peaks from three independent biological replicates. Data for Mn was not included as the majority of cells measured were found to be below the limit of detection and no clear distributions could be observed

*M. tuberculosis* primary intracellular niche within a human host is within macrophages, where trace element concentrations impact the host immune response, bacterial/host metabolism, and bacterial survival. Therefore, understanding this interaction requires sensitive methods to accurately detect and quantify trace metals [31]. Until now, these measurements have been made on bulk populations yielding average data, missing important heterogeneity, which can impact disease progression and antibiotic treatment [2–4, 26, 27]. Here, we have developed an approach to apply SC-ICP-MS to a macrophage model of tuberculosis (TB). In order to assess effective removal of adherent cells with minimal rupture, three processes were tested. Dislodging the cells through cell scraping and PFA fixation allowed single infected cells to be analysed. This method was compared to enzymatic removal by trypsin (resulting in the recovery of 3.5 × 10^6^ cells compared to 6.1 × 10^6^ cells with the scraping and then fixing method) and fixation prior to scraping (high levels of clumping observed by microscopy). We also used a fluorescent reporter strain of *M. bovis* BCG (Fig. SI 6) so that we could apply flow cytometry to measure the level of infection. Approximately 25% cells were infected at the point of introduction into the ICP-MS in accordance with infection efficiency of PMA-induced THP-1 cells [40]. SC-ICP-MS was used to measure the elemental composition of infected and uninfected THP-1 macrophages, elemental masses were calculated and the relative probability densities for each population of cells were compared (Fig. 6). The probability densities indicate the relative probability (*y*-axis, arbitrary value) that a variable will fall at a specified value (*x*-axis, mass in fg). Preliminary results indicate that calcium levels were tightly regulated, and very little heterogeneity was measured in the amount of this metal in monocytes and macrophages and also within our TB macrophage model.

Similarly for zinc, there was also little variation between uninfected and infected macrophages whereas monocytes showed significant heterogeneity indicating that immune activation tightly regulates zinc levels. However, mycobacterial infection led to significant heterogeneity in the levels of magnesium. This is significant as magnesium has a variety of biological functions in regulating energy metabolism, enzyme activity, signal transduction, nucleic acid and protein synthesis and is known to be immunomodulatory and shown to drive an anti-inflammatory response (M2 phenotype) [41]. This observed heterogeneity could therefore have significant effects on the course of mycobacterial infection and potentially impact on antimicrobial susceptibility.

## Conclusion

This work has shown that direct analysis of live eukaryotic cells using SP-ICP-MS is unfeasible due to the high background caused by the complex cell culture media, and therefore, fixation and resuspension are essential. This work shows that selection of fixatives is not trivial and can significantly impact the analysis of trace metals. MeOH fixation (60–100%) resulted in significant leaching of Mg and Ca, with less severe effects observed for Mn and Zn when compared to PFA fixation. Using our method, we successfully measured semi-quantitatively trace elements in THP-1 monocytes and infected and uninfected macrophages demonstrating significant heterogeneity that would have been invisible by bulk analysis. This approach can now be applied to study host–pathogen interactions and other important biological phenomena using SC-ICP-MS.

## Supplementary Information

Below is the link to the electronic supplementary material.

Supplementary file1 (DOCX 2855 KB)
