# Supplementary material for: Single cell–inductively coupled plasma–mass spectrometry (SC-ICP-MS) reveals metallic heterogeneity in a macrophage model of infectious diseases
Source: Anal Bioanal Chem. 2024 Oct 17;416(29):6945–55. doi: 10.1007/s00216-024-05592-3 (PMC11579058; doi:10.1007/s00216-024-05592-3)
Supplement: Supplementary file 1 — Supplementary file1 (DOCX 2855 KB) [file 216_2024_5592_MOESM1_ESM.docx]

**Supporting information**

**Single cell – inductively coupled plasma – mass spectrometry (SC-ICP-MS) reveals metallic heterogeneity in a macrophage model of infectious diseases**

**Claire Davison^1,2^, Jordan Pascoe^2^, Melanie Bailey^1^, Dany JV Beste^2^ and Mónica Felipe-Sotelo^1*^.**

^1^School of Chemistry and Chemical Engineering, Faculty of Engineering and Physical Sciences,
University of Surrey, Guildford, UK

^2^ Department of Microbial Science, Faculty of Health and Medical Sciences, University of Surrey, Guildford, UK

*Corresponding author: m.felipe-sotelo@surrey.ac.uk. Contributing authors: claire.davison@surrey.ac.uk; jp00237@surrey.ac.uk; m.bailey@surrey.ac.uk; d.beste@surrey.ac.uk

ORCID: 0000-0001-6120-4451 (CD), 0000-0002-4373-1829 (JP), 0000-0001-9050-7910 (MB), 0000-0001-6579-1366 (DB), 0000-0002-8030-7648 (MFS)

**Instrumental conditions and optimisation of SC-ICP-MS analysis: Calculation of transport efficiency (TE%) and mass content**

Transport efficiency (TE) is defined as the ratio of the analyte entering the detector to the amount of analyte aspirated and is calculated using a suspension of known size and/or particle number concentration [1]. Pace et al. (2011) [1] described in detail and compared transport efficiency calculation approaches, specifically the particle size method and the particle frequency method.

Transport efficiency is an essential parameter for full quantification of single cells using SC-ICP-MS, however selection of transport efficiency calculation approach should be based on the objective of the analysis. The particle size method estimates TE as a fraction of the total mass introduced into the instrument compared to that which is measured. This is ideal when full quantitative measurements is the objective of the study as it accounts for loss of mass. However, available particle reference materials are often limited to gold and silver. Analytes behave differently upon introduction into the ICP and to accurately apply this method element specific corrections are required. On the contrary, the pulse frequency method determines TE by assessing whole particle introduction rather than mass. The advantage here is that real samples can be used to estimate TE rather than reference materials which are often not of comparable size and/or composition to the particle being analysed. This is most useful for the optimisation of sample introduction and instrumental operating conditions as it is the most accurate representation of the behaviour of the real particles [2].

There are two potential approaches to the application of SC-ICP-MS: (i) Absolute quantifications of metals at single cell level or (ii) comparative analysis of cell populations under different biological conditions. In the 1^st^ case for full quantification, it is necessary to have an accurate estimation of the TE% in order to carry out the mass calculations and to maximize this parameter. However, in the case of comparative analysis, such is the case of this manuscript (MeOH vs PFA; infected vs non-infected) the factor that was prioritised was to maximise the number of detected peaks in order to obtain population of measured cells that were statistically significant when compared to other populations measured under the same conditions. For this reason, the pulse frequency method (as described by Pace et al. (2011) [1]) was used here by measuring a cellular suspension of known particle number concentration (assessed using a haemocytometer).

$$Transport efficiency=\frac{Number of intensity peaks produced by single cells (cells/ms)}{Sample flow rate (ml/ms) x concentration of cells (cells/ml)}$$

Once transport efficiency has been determined, a dissolved standard calibration curve was prepared. Signal intensity was then correlated to total analyte mass entering the plasma for each standard, as described in detail by Pace et al. (2011) [1]).

$$Mass per event \left( \mu g \right)$$

$$=TE x sample flow rate (ml/ms) x dwell time (ms/event) x analyte concentration (\mu g/ml)$$

The blank corrected intensity signal was then inserted into the transformed calibration curve (y-axis intensity, x-axis mass per event) to determine the mass of each single cell event.

**References in this Supplementary Information**

[1] Pace HE, Rogers NJ, Jarolimek C, Coleman VA, Higgins CP, Ranville JF (2011) Determining Transport Efficiency for the Purpose of Counting and Sizing Nanoparticles via Single Particle Inductively Coupled Plasma Mass Spectrometry. Anal Chem 83:9361–9369. https://doi.org/10.1021/ac201952t

[2] Davison C, Beste D, Bailey M, Felipe-Sotelo M (2023) Expanding the boundaries of atomic spectroscopy at the single-cell level: critical review of SP-ICP-MS, LIBS and LA-ICP-MS advances for the elemental analysis of tissues and single cells. Anal Bioanal Chem. https://doi.org/10.1007/s00216-023-04721-8

**Tables SI 1-5**

**Table SI 1** SC-ICP-MS standard operating conditions.

| **Instrument** | |
| --- | --- |
| Instrument | 7800 Series ICP-MS (Agilent Technologies, UK) |
| Sample introduction | Manual |
| Sample pump tube (mm) | 1.02 |
| ICP Torch injector (internal ø mm) | 1.5 |
| Nebuliser | Concentric Micro Mist (G3266-80004) |
| Spray Chamber | Quartz Scott Double Pass |
| Daily Tune | Li, Y, Ce, Tl and Co in 2% HNO_3_ 10 ng L^-1^  (Agilent Technologies, UK) |
| **Plasma operating conditions** | |
| Plasma Mode | Low Matrix; No Gas |
| RF Power (kW) | 1.55 |
| Nebuliser gas flow (L min^-1^) | 0.7 |
| **Flow parameters** | |
| Nebuliser pump rate (mL min^-1^) | 1.67 |
| Uptake time (s) | 30 |
| Stabilisation time (s) | 100 |
| Wash time (s) | 120 |
| Wash | Deionised water |
| Integration time (s) | 0.003 |
| Measurement time (s) | 60 |

**Table SI 2** Replicate cell counts of a PFA fixed THP-1 suspension to assess stability of cell count using microscopy and hemocytometry at concentrations ranging from approximately 5.0 × 10^3^ to
5.0 × 10^6^ cells mL^-1^.

| **Cell suspension** | **Average count (cells mL^-1^, n=3)** | |
| --- | --- | --- |
|  | **T = 0 h** | **T = 12 h** |
| **A** | 4.9 × 10^6^ ± 0.3 × 10^6^ | 4.8 × 10^6^ ± 0.7 × 10^6^ |
| **B** | 1.1 × 10^6^ ± 0.1 × 10^6^ | 1.0 × 10^6^ ± 0.1 × 10^6^ |
| **C** | 4.9 × 10^5^ ± 0.5 × 10^5^ | 5.1 × 10^5^ ± 0.8 × 10^5^ |
| **D** | 1.7 × 10^5^ ± 0.4 × 10^5^ | 1.6 × 10^5^ ± 0.2 × 10^5^ |
| **E** | 6.7 × 10^4^ ± 1.5 × 10^4^ | 6.7 × 10^4^ ± 0.6 × 10^4^ |
| **F** | 2.3 × 10^4^ ± 0.6 × 10^4^ | 2.0 × 10^4^ ± 0.3 × 10^4^ |
| **G** | 5.0 × 10^3^ ± 2.5 × 10^3^ | 5.8 × 10^3^ ± 1.4 × 10^3^ |

**Table SI 3** Replicate cell counts of a high and low concentration THP-1 suspension to assess stability of 70% MeOH and 4% PFA fixation (n=3).

| **Fixation method** | **Average count (cells mL^-1^, n=3)** | |
| --- | --- | --- |
|  | **T = 0h** | **T = 12h** |
| **MeOH** | 4.6 × 10^5^ ± 0.2 × 10^5^ | 4.5 × 10^5^ ± 0.1 × 10^5^ |
| **PFA** | 4.8 × 10^5^ ± 0.2 × 10^5^ | 4.8 × 10^5^ ± 0.5 × 10^5^ |

**Table SI 4** Elemental analysis of solvents used to fix THP-1 cells to determine the occurrence of a leaching effect in Mg, Ca, Mn and Zn (n=3).

| **Fixation method** | **Concentration (ng L^-1^)** | | | |
| --- | --- | --- | --- | --- |
|  | **Mg** | **Ca** | **Mn** | **Zn** |
| **60% MeOH** | 638.3 ± 7.8 | 871.5 ± 7.3 | 7.3 ± 0.04 | 27.4 ± 0.7 |
| **80% MeOH** | 793.3 ± 16.0 | 1043.1 ± 6.3 | 8.2 ± 0.04 | 20.9 ± 0.6 |
| **100% MeOH** | 193.0 ± 3.0 | 212.9 ± 8.5 | 4.7 ± 0.005 | 6.2 ± 0.4 |

**Table SI 5** Comparison of average single cell measurements using SC-ICP-MS (PFA fixation and particle frequency method using THP-1 cells) and bulk measurement of microwave digested cells using solution ICP-MS (n=3, ± SD).

|  | **Total mass per cell (fg)** | |
| --- | --- | --- |
|  | **SC-ICP-MS** | **Bulk measurement (n=3, ± SD)** |
| **Mg** | 0.55 | 67 ± 4 |
| **Ca** | 2.54 | 357 ± 7 |
| **Zn** | 0.55 | 29 ± 11 |
| **Mn** | 0.12 | 0.9 ± 0.1 |

**Figures SI 1-5**

**Figure SI 1** Cell number concentration (cells / mL) plotted against the background Mg^24^ signal (counts). Error bars represent ± 1 x SD (n=6).

**Figure SI 2 (a)** Sample flow rate (mL min^-1^) plotted against the Mg^24^ intensity cut-off for a single cell (counts) **(b)** Sample flow rate (mL min^-1^) plotted against the maximum intensity for a single cell (counts). Error bars represent ± SD (n=3).

**Figure SI 3** Direct time resolved SC-ICP-MS analysis of ^24^Mg in THP-1 cells in culture media
(RPMI Media + 10% FCS).

**Figure SI 4** Time resolved SC-ICP-MS analysis of ^24^Mg in THP-1 cells (70% MeOH fixation).

**Figure SI 5** Time resolved SC-ICP-MS analysis of ^24^Mg in THP-1 cells (4% PFA fixation).

**
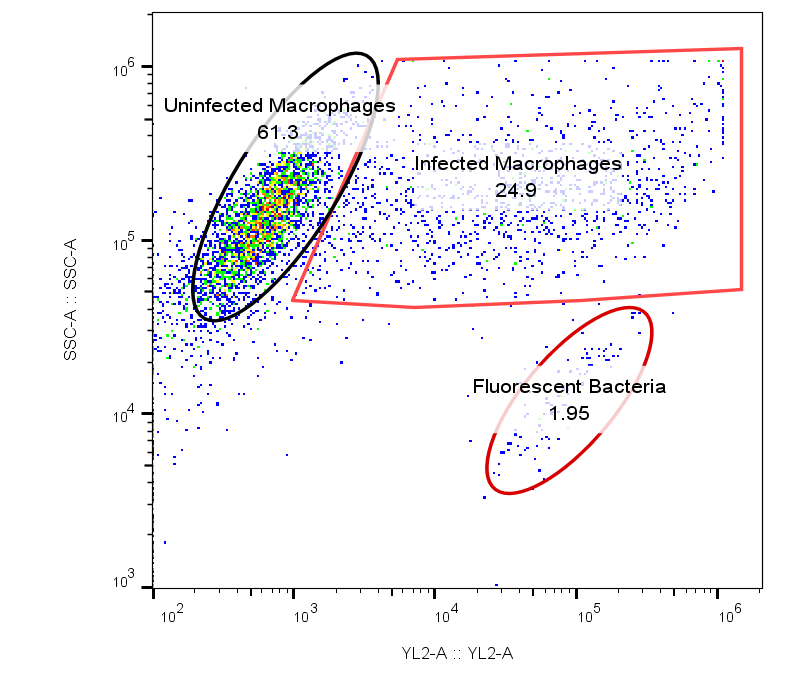
**

**Figure SI 6** Gating strategy employed for determining the percentage of PMA induced THP1s that were infected with *Mycobacterium bovis* Bacillus Calmette–Guérin (BCG)
